# Filter's retraction hook capture technique of pull-assisted method for endovascular retrieval of conical inferior vena cava filters whose hook attached to the wall

**DOI:** 10.3389/fsurg.2026.1674195

**Published:** 2026-01-22

**Authors:** Xuan Tian, Jianlong Liu, Han Zheng, Jinyong Li, Xiao Liu, Mi Zhou, Chengjia Qu, Run Hua, Chenyang Tian

**Affiliations:** 1 Vascular Surgery Department, Beijing Jishuitan Hospital, Capital Medical University, Beijing, China; 2 Capital Medical University, Beijing, China

**Keywords:** advanced retrieval techniques, biopsy forceps, filter retrieval, pulmonary embolism, vena cava filter

## Abstract

**Background:**

Permanent placement of venous filters can lead to numerous complications. When the risk of pulmonary embolism (PE) decreases, it is recommended to retrieve the filter. Inferior vena cava (IVC) filter retrieval is primarily performed intraluminally; however, the retrieval hook for conical filters may penetrate the venous wall, causing failure of the intraluminal retrieval: some filters are retrieved using the Loop-snare technique or its modified version, some are retrieved through open surgery, which causes more damage, and some are left permanently in place. For these patients, a filter's retraction hook capture technique of pull-assisted method can be used effectively to retrieve the filter. This study introduces a surgical method using the novel technique for the intraluminal removal of conical IVC filters whose retraction hook attached to the wall, along with the outcomes and a 3-month follow-up.

**Methods:**

From January 2021 to December 2024, patients with conical filters whose retraction hook attached to the wall were enrolled consecutively. Various advanced filter retrieval techniques were initially used to remove the filters, and those that were unsuccessful were subsequently treated with the new technology for filter retrieval. The patients were divided into a successful group and a failure group based on whether the filter retrieval was successful. Retrospective comparative analysis was performed to evaluate patient characteristics, filter retrieval rate, inclination, penetration distance, and IVC imaging.

**Results:**

A total of 44 patients underwent filter retrieval using filter's retraction hook capture technique of pull-assisted method. Among these patients, 37 cases (84.1%) were successful in filter retrieval (successful group), with the penetration distance of cranial anchor vertex of 3.2 (2.5, 4.3) mm, and 12 (32.4%) filters were deformed. The other seven cases (failure group) were unsuccessful, with a penetration distance of cranial anchor vertex of 5.0 (4.3, 5.0) mm, and 6 (85.7%) filters were deformed. There was a statistically significant difference between the two groups (P < 0.05). One case (2.3%) had IVC injury, one case (2.3%) experienced filter fracture, and no symptomatic PE occurred. Logistic regression analysis was performed to identify factors that might affect filter retrieval, with an odds ratio (OR) of 0.069 (0.006, 0.828), suggesting a statistical difference between filter deformation and successful retrieval. Logistic regression analysis was also performed to determine factors influencing filter inclination, with the results indicating a statistically significant difference in the penetration distance and the transverse diameter of the IVC [OR = 0.667 (0.465, 0.958) and OR = 0.843 (0.712, 0.998), respectively], indicating a statistically significant difference in the penetration distance and the transverse diameter of the IVC, and affecting severe filter inclination.

**Conclusion:**

Filter's retraction hook capture technique of pull-assisted method is effective in removing conical filters whose hook attached to the wall, with no symptomatic PE occurring. This method can be considered as a new adjuvant technique for filter retrieval.

## 1 Introduction

Permanent placement of filters may lead to numerous complications (1–3). Therefore, guidelines recommend (4–9) the placement of retrievable inferior vena cava filters (IVCF) during the perioperative period, to be retrieved once the risk of pulmonary embolism (PE) decreases. In China, conical filters, which are characterized by point contact with the venous wall and a long dwell time, began to be used in 2013 for patients with acute deep vein thrombosis and are widely used in clinical practice; however, they are prone to tilting, which can cause the retrieval hook to penetrate the inferior vena cava (IVC) wall, leading to failure of intraluminal retrieval of the filter (10).

The filter retrieval rate in reality is still quite low (11, 12), with only 40%–76.9% filters were retrieved after a placement for one year. The factors of failure for filter retrieval using the standard technique mainly includes: filter embedded in IVC wall, filter angulation (>15°), significant leg penetration (more than 15 mm), prolonged dwell time (due to lack of follow-up) (31). For conical filters, the main factors of failure for filter retrieval using the standard technique includes severe filter inclination, the filter's retraction hook penetrating through the wall of the IVC, which often needs to use advanced technique to enhance the filter retrieval rate.

When the retrieval hook of a conical filter penetrates the IVC wall, the following methods can be used to remove the filter: (1) The Loop-snare technique corrects the filter inclination (13, 14), and the key application feature of this technique is to retrieve the filter after letting the filter coaxially align with the retrieval sheath; (2) The modified Loop-snare technique cuts the hook's endothelial tissue, allowing the hook to re-enter the IVC (15, 16), which allows for the capture of the filter's retrieval hook, thereby retrieving the filter; (3) The Biopsy forceps directly pull the filter into the retrieval sheath (15); (4) The catheter push technique removes the filter (17), which is suitable for hollow conical filters. After the guidewire passes through the hollow lumen, its two sides would correct the filter inclination, retrieving the filter after capturing its filter retrieval hook. But when the filter's retraction hook attaches to the wall, the hollow lumen would be filled by endothelial tissues, making it difficult for the guidewire to pass through; (5) Laparoscopic filter removal (18), which is suitable for conical filters of which retrieval hook has already completely penetrated the wall of IVC, and is particularly appropriate when the hook has penetrated the anterior or lateral wall of the IVC; (6) Open abdominal filter removal (19, 20), which is suitable for conical filters which already develop complications such as fracture, and of which the retrieval hook has completely penetrated the posterior wall of the IVC. However, the following situations may occur: after using the Loop-snare and modified Loop-snare techniques, the filter still cannot be removed; the Biopsy forceps method may risk damaging the vein; laparoscopic or open abdominal surgery to remove the filter carries a greater risk of iatrogenic injury than intraluminal retrieval of the filter.

For conical filters that could not be removed after using the two Loop-snare techniques, the filter's retraction hook capture technique of pull-assisted method can be used to remove the filter. This article primarily introduces the effectiveness, surgical method, and outcome analysis of using the filter's retraction hook capture technique of pull-assisted method for the intraluminal removal of conical IVCFs with a retrieval hook that has penetrated the IVC wall.

## 2 Materials and method

### 2.1 General information

**Figure 1:**
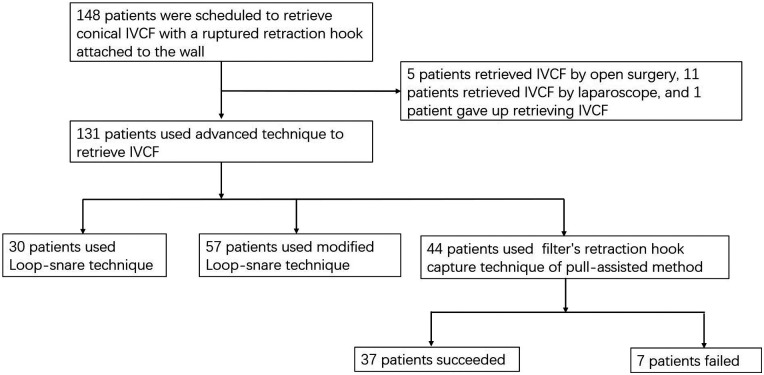
Flow chart of patients who choose method of retrieving IVCF.

A retrospective analysis was conducted on patients from January 2021 to December 2024 who had conical filters with retrieval hooks attached to the IVC wall. Advanced filter retrieval technologies were used, including the Loop-snare technique, modified Loop-snare technique (Hangman technique), and traction-assisted retrieval hook technology. The specific situations are detailed in Table 1 and Figure 1. This study was conducted with the approval and informed consent of the enrolled patients.

**Table 1:** General condition and perioperative status form.

Topic	Success Group	Failure Group	Z/t	*P*	IVC Filter Retrieval Using Different Methods in the Success Group			Z/t	*P*
					Loop-snare	Hangman	New technique		
Case Count(*n*)	37 (84.1)	7 (15.9)	4.372	<0.001	12 (32.4)	7 (18.9)	18 (48.6)	4.919	−0.085
Age (years)	56.1 ± 13.7	45.9 ± 15.1	3.177	0.075	52.3 ± 13.8	65.0 ± 6.7	55.1 ± 14.6	4.425	0.109
Gender (male)	27 (73.0)	5 (71.4)	1.000	0.628	8 (66.7)	4 (57.1)	15 (83.3)	2.111	0.348
Height (cm)	168.4 ± 8.3	171.3 ± 5.0	1.030	0.310	169.3 ± 8.1	163.3 ± 8.3	169.8 ± 8.1	4.132	0.127
Weight (kg)	69.6 ± 11.2	69.6 ± 12.9	0.000	0.987	70.7 ± 7.4	66.1 ± 10.1	70.1 ± 13.8	0.497	0.780
Bodymassindex(BMI)	24.5 ± 3.4	23.6 ± 3.6	0.315	0.574	24.8 ± 3.4	24.8 ± 3.1	24.2 ± 3.7	0.731	0.694
Indwelling Time(d)	100.0 (60.0, 162.5)	180 (50,190)	0.335	0.563	110.0 (66.0, 153.8)	100 (90,180)	96.0 (57.5, 168.8)	0.419	0.811
Inclination a Angel(°)	11.9 (8.3, 18.6)	11.6 (7.9,27.0)	0.001	0.974	14.6 (9.2,20.9)	8.4 (6.1,18.0)	11.2 (8.0,18.5)	3.902	0.412
Severe Inclination	12 (32.4)	2 (28.6)	0.040	0.841	5 (41.7)	2 (28.6)	5 (27.8)	0.693	0.707
Penetration Distance of Cranial Anchor Vertex(mm)	3.2 (2.5, 4.3)	5.0 (4.3,5.0)	6.027	0.014	3.3 (2.5, 6.6)	3.4 (2.5, 4.4)	3.0 (2.4,3.9)	0.586	0.746
>3 mm Vessel Wall Transgression(*n*)	19 (51.4)	7 (100)	5.763	0.031	7 (58.3)	3 (42.9)	9 (50)	0.449	0.799
Transverse Diameter of IVC(mm)	21.0 ± 5.1	21.2 ± 4.8	0.013	0.911	22.4 ± 4.0	18.5 ± 3.9	21.1 ± 6.0	3.397	0.183
Longitudinal Diameter of IVC(mm)	14.9 ± 3.4	14.1 ± 3.9	0.352	0.553	14.9 ± 2.3	14.6 ± 3.8	15.1 ± 4.0	0.343	0.842
Transverse + Longitudinal Diameter(mm)	36.0 ± 7.0	35.3 ± 6.0	0.013	0.911	37.3 ± 5.3	33.1 ± 6.2	36.2 ± 8.3	2.057	0.358
Mean(mm)	18.0 ± 3.5	17.6 ± 3.0	0.013	0.911	18.7 ± 2.6	16.5 ± 3.1	18.1 ± 4.1	2.057	0.358
Penetration Distance of Acudal Anchor Tip(mm)	3.8 (2.7, 5.3)	4.4 (3.3, 8.0)	1.526	0.217	3.5 (2.5, 5.0)	3.4 (3.2, 5.9)	4.0 (2.2,7.0)	0.383	0.826
Radiation Dose(Gy)	301.0 (172.0, 557.5)	292.0 (214.0, 755.0)	0.247	0.619	352.5	172 (127, 1270)	293.0	0.146	0.930
Prior Retrieval Attempts(*n*)	1 (1,1)	1 (1,2)	0.179	0.672	(113.8, 572.8)	1 (1,1)	(207.3, 498.3)	1.600	0.449
Vascular Access Route for IVC Implantation (Right Femoral)	17 (45.9)	5 (71.4)	0.412	0.206	1 (1,1)	3 (42.9)	1 (1,1)	0.234	0.889
IVC Deformation(*n*)	12 (32.4)	6 (85.7)	0.042	0.036	5 (41.7)	2 (28.6)	9 (50)	3.302	0.192
Filter Brand(*n*)	12 (32.4)	1 (14.3)	2.254	0.689	3 (25.0)	0	7 (38.9)	10.578	0.227
Denali(BD)	5 (13.5)	1 (14.3)			5 (41.7)	2 (28.6)	9 (50)		
Celect(Cook)	9 (24.3)	2 (28.6)			1 (8.3)	4 (57.1)	2 (11.1)		
Option(Argon)	8 (21.6)	3 (42.9)			3 (25.0)	1 (14.3)	2 (11.1)		
Octoparms®	1 (2.7)	0			3 (25.0)	0	4 (22.2)		
Others					0		1 (5.6)		

### 2.2 Inclusion criteria

Patients aged < 80 years, with no contraindications for anticoagulation, and no severe cardiopulmonary disease that would prevent them from tolerating surgery; patent IVC and at least one patent iliac vein, and no thrombosis or only a small amount of old thrombosis in the lower extremity veins and IVC, with a low risk of PE after filter removal; preoperative CT showing severe inclination of the conical filter with the retrieval hook penetrating the IVC wall; patients unwilling to leave the filter permanently in place and who strongly desire retrieval; and patients who underwent retrieval using the traction-assisted retrieval hook technology.

### 2.3 Grouping method

Patients who successfully had the filter retrieved using filter's retraction hook Biopsy technique of pull-assisted method were placed in the successful group, while those in whom the filter was not retrieved using this technology were placed in the failure group.

### 2.4 Treatment method

#### 2.4.1 Preoperative examination

Ultrasound of the lower extremity veins and IVC was performed to observe IVC patency, patent iliac veins (bilateral or at least one side), and the presence of thrombosis or old thrombosis in the lower extremity veins, with a low risk of thrombus dislodgement. Abdominal enhanced CT was used to check the patency of the IVC, whether the retrieval hook had penetrated the vessel wall (21), and to measure the distance from the filter tip to the IVC wall, the inclination angle of the filter, the distance from the pedicle to the IVC wall, the transverse and longitudinal diameters of the IVC. PE symptoms in the observed patients were also assessed.

#### 2.4.2 Main measurement indicators

All imaging data were uploaded to the Carestream imaging workstation and independently assessed and measured by two vascular surgeons who were not involved in the patient's clinical management or the retrieval procedure. To minimize measurement error, each measurement was repeated three times and the mean value was used for analysis. If the calculated results differed by >1.5 mm between the two assessors, a third independent assessor (a vascular surgeon or radiologist) was invited to repeat the measurement. The two closest values were then averaged and used as the final result.

The penetration distance of the filter tip is defined as the distance from the retrieval hook tip to the IVC wall, measured during the enhanced CT venous phase. Some studies suggest that when the distance exceeds 3 mm, the vein wall is considered to be penetrated (22).

The tilt angle is defined as the angle between the line connecting the filter cranial anchor vertex to the midpoint of the diagonal of the pedicles and the longitudinal axis of the IVC. An inclination angle greater than 15 degrees is considered as severe inclination (16).

On the transverse section of the enhanced CT, the IVC is typically elliptical in shape. The transverse diameter refers to the horizontal diameter of the IVC, and the longitudinal diameter refers to the vertical diameter of the IVC.

The penetration distance of the filter acudal anchor tip is defined as the distance from the tip of each pedicle to the IVC wall, measured during the enhanced CT venous phase, with the maximum value taken.

Filter deformation is defined as the presence of filter breakage, upward bending or displacement of the pedicles or accessory wires.

#### 2.4.3 Surgical procedure

First, the Loop-snare technique and the modified Loop-snare technique (Hangman technique) are used for filter retrieval. The Loop-snare technique (14) involves forming a wire loop inside the filter and pulling to correct the filter's tilt angle. If unsuccessful, the modified Loop-snare technique (16) is used, which forms a wire loop between the filter neck and the IVC wall. Under the reverse force between the guidewire (Terumo angled, 260 cm) and the retrieval sheath (BD, Snare Retrieval Kit), this technique cuts and destroys the proliferated tissue surrounding the retrieval hook, making the hook free within the IVC. When both Loop-snare techniques fail, the filter's retraction hook capture technique of pull-assisted method is attempted.

The procedure enters through the femoral vein pathway, with the filter retrieval sheath (it is recommended to use an adjustable curved sheath—Lifetech, SVA10F-550—which can adjust the direction of the biopsy forceps) and biopsy forceps. The forceps capture the filter's main pedicle (avoiding the capture of accessory wires or balancing arms) and pull the filter toward the distal end. It is recommended to clamp the pedicle in the opposite direction of the inclination of the retrieval hook, as this not only moves the filter towards the distal end but also reduces the filter's inclination angle. While pulling the filter toward the distal end, the proximal end can be retrieved using the following methods: (1) Snare: When pulling the filter toward the distal end, it can reduce the inclination angle of the filter, providing an opportunity to directly release the endothelial tissue of the retrieval hook. Once exposed, the snare can directly capture it; (2) Loop-snare technique: When the Biopsy forceps clamp the pedicle in the opposite direction of the inclination of the retrieval hook, the Loop-snare technique can cut the endothelial tissue attached to the retrieval hook and successfully retrieve the filter; (3) Modified Loop-snare technique: The principle of this method is the same as the Loop-snare technique. The traction exposes the gap between the filter neck and the IVC wall, forming a wire loop; (4) Biopsy forceps: The Biopsy forceps directly clamp the retrieval hook and retrieve the filter (same method as above) (23). For retrieval hooks with endothelial tissue covering the surface, the Biopsy forceps can destroy and separate the endothelial tissue, thus exposing the retrieval hook.

**Figure 2:**
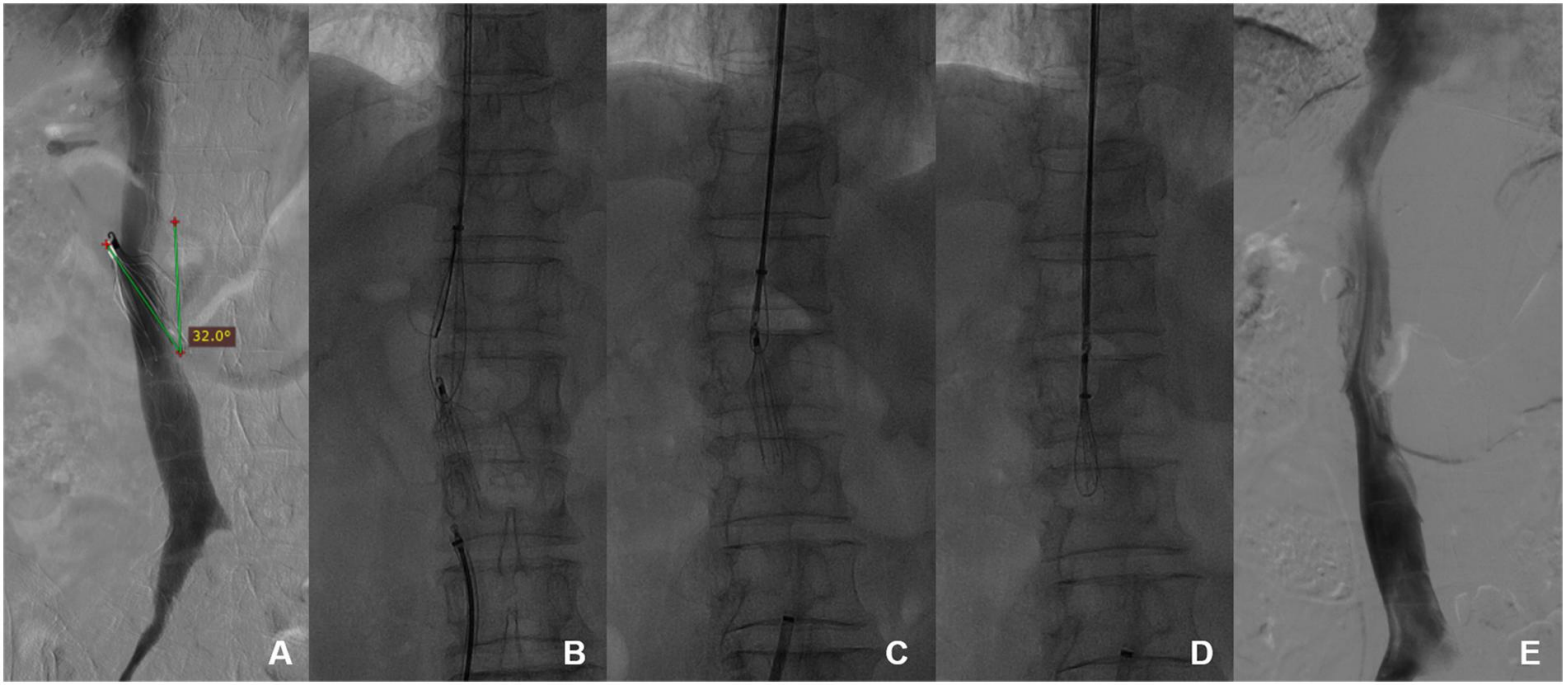
Use of the filter's retraction hook capture technique of pull-assisted method to retrieve a severely inclined conical filter **(A)**. The conical filter has a severe inclination angle of 32°. **(B)** The distal end is pulled with a snare, and advanced retrieval techniques are attempted at the proximal end. **(C)** The proximal end uses forceps to capture the filter retrieval hook. **(D)** The snare successfully retrieves the filter. **(E)** No damage is seen on inferior vena cava (IVC) angiography.

For patients in whom intraluminal retrieval fails but still require filter retrieval, laparoscopic filter retrieval or open abdominal filter retrieval may be chosen. If the patient refuses more invasive surgery, the filter may be permanently left in place (Figure 2).

The procedure in this study was required to be performed by vascular surgeons holding senior professional titles.

#### 2.4.4 Postoperative treatment

Low molecular weight heparin 100 IU/Kg Q12h for anticoagulation therapy. Upon discharge, oral rivaroxaban 20 mg QD for anticoagulation therapy (24), and plasma D-dimer monitoring is performed one month later.

Postoperative 3-month follow-up involved ultrasound examination of the lower extremity veins to observe changes in limb thrombosis and enhanced abdominal CT to check IVC patency. PE symptoms were also monitored. After discontinuing anticoagulation therapy for 3 months, ultrasound of the lower extremities and IVC was repeated to observe IVC patency and changes in limb thrombosis.

### 2.5 Statistical analysis

SPSS 27.0 software was used for statistical analysis. Continuous data are expressed as mean ± standard deviation; categorical data are described as percentages; non-normally distributed continuous data are presented as [M (P25, P75)]; the Wilcoxon rank-sum test was used for group comparisons, with a *P* value < 0.05 considered statistically significant. Logistic regression analysis was conducted to assess factors influencing filter retrieval and tilt, and a two-tailed *P* value < 0.05 was considered statistically significant.

## 3 Results

### 3.1 Regarding the study population

A total of 148 patients were scheduled to retrieve conical IVCF with a ruptured retraction hook attached to the wall (Figure 1), of which 141 (95.3%) were transferred from outside hospitals. 131 patients (88.5%) used advanced technique to retrieve IVCF, 5 patients (3.4%) retrieved IVCF by open surgery, 11 patients (7.4%) retrieved IVCF by laparoscope, and 1 patient (0.7%) gave up retrieving IVCF (Table 1).

A total of 44 patients (29.8%) used the filter's retraction hook capture technique of pull-assisted method for filter retrieval. Among these, 37 patients (84.1%) succeeded, and 7 patients failed. Of the failed cases, 3 patients (42.9%) underwent open abdominal retrieval, 3 patients (42.9%) underwent laparoscopic retrieval, and 1 patient (14.2%) refused further retrieval surgery because the filter was located in the renal IVC and refused further surgical intervention.

### 3.2 Surgical outcomes

Among the 44 patients, 37 successfully had the filter removed, resulting in an 84.1% success rate for the removal of conical filters whose retraction hook attached to the wall. In the successful group, the penetration distance of cranial anchor vertex was 3.2 (2.5, 4.3) mm, with 12 cases (32.4%) of filter deformation (Z = 6.027 and *P* = 0.014). In the failed group, the penetration distance of cranial anchor vertex was 5.0 (4.3, 5.0) mm, with 6 cases (85.7%) of filter deformations (X^2^ = 0.042 and *P* = 0.036). There was a statistical difference. Regarding inclination angle and indwelling time, the successful group values were respectively 11.9 (8.3, 18.6) and 100.0 (60.0, 162.5) days, and the unsuccessful group values were respectively 11.6 (7.9, 27.0) and 180 (50, 190) days, with a *Z* value respectively 0.001 and 0.335, a *P* value respectively 0.974 and 0.563. For the traverse diameter of IVC, the successful group measured 21.0 ± 5.1 mm, and the unsuccessful group measured 21.2 ± 4.8 mm, with *F* = 0.013 and *P* = 0.911, demonstrating no statistical difference (Table 1). There is no correlation between whether the conical filters attaching to the wall were retrieved and the indwelling time.

**Figure 3:**
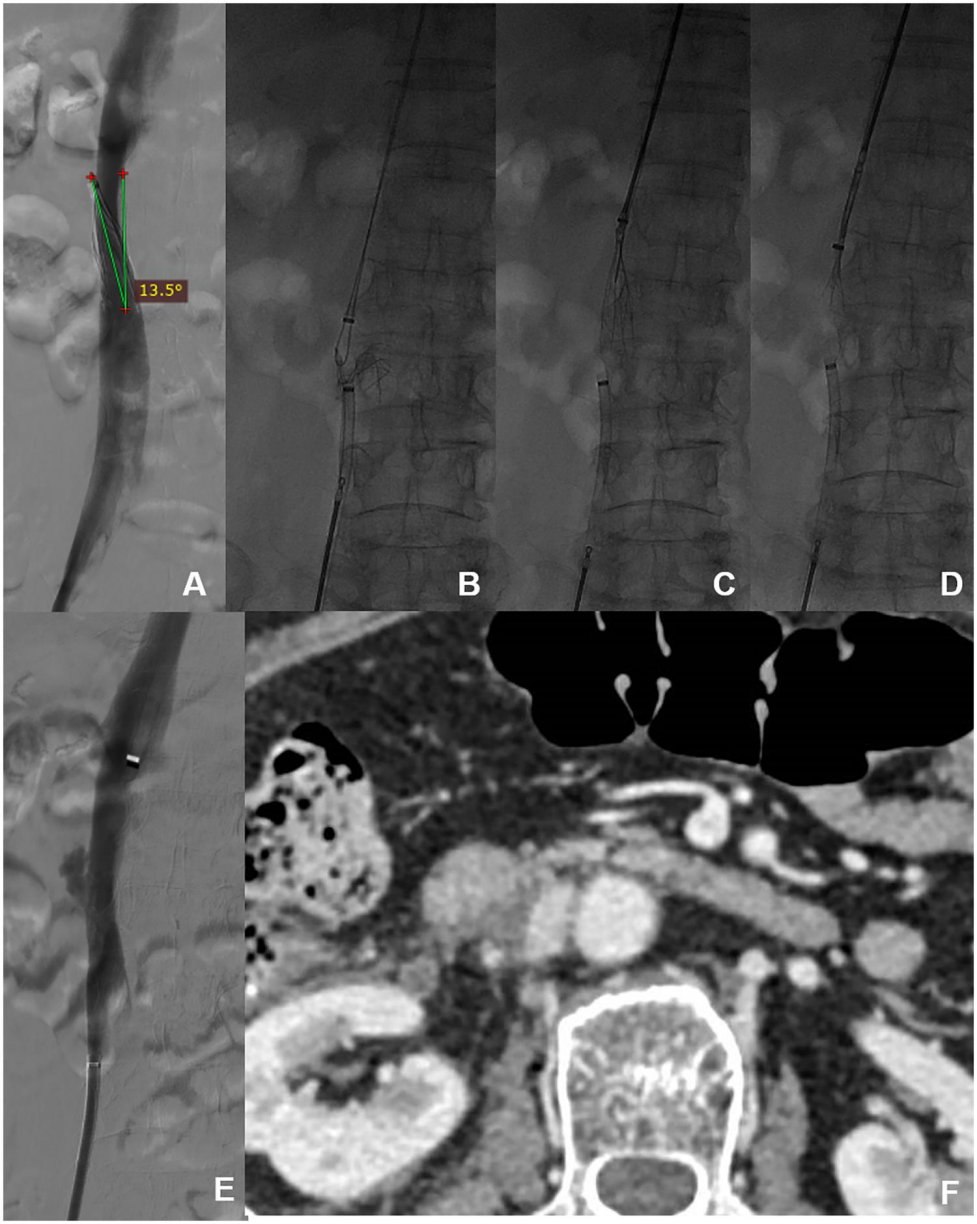
Complications during the filter's retraction hook capture technique of pull-assisted method for tilted wall-attached conical filter. **(A)** The conical filter has a tilt angle of 13.5°. **(B)** The distal end is pulled with a snare, and advanced retrieval techniques are attempted at the proximal end. **(C)** The proximal end uses forceps to capture the filter retrieval hook. **(D)** The snare successfully retrieves the filter. **(E)** IVC damage is observed. **(F)** Postoperative enhanced CT scan of the IVC one week after Interventional surgery.

**Figure 4:**
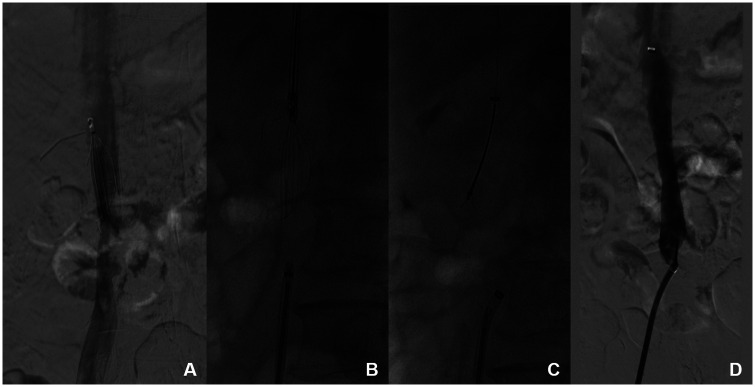
Retrieval of a conical filter with a broken filter strand using the filter's retraction hook capture technique of pull-assisted method **(A)**. Wall-attached conical filter with a broken filter strand. **(B)** The distal end is pulled with a snare, and advanced retrieval techniques are attempted at the proximal end. **(C)** The snare captures the broken filter strand and removes it in the lumen. **(D)** No damage is seen on IVC angiography.

One patient experienced IVC damage, with no abnormal vital signs, and after conservative treatment and 1 week of bed rest, a follow-up CT scan showed stenosis at the damaged IVC site with thrombosis attached, but no IVC obstruction (Figure 3). One patient was found to have a broken filter strand. After the main filter was removed, a snare was successfully used to retrieve the broken strand (Figure 4). No symptomatic PE occurred.

Among the 37 patients with successful filter removal, the methods used to pull the filter distally and retrieve it proximally during surgery were as follows: 12 patients (32.4%) had filters removed using the Loop-snare technique, 7 patients (18.9%) had filters removed using the Hangman technique, and 18 patients (48.6%) had filters removed using the snare technique, with X^2^ = 4.919 and *P* = 0.085. There was no statistical difference.

During the 3-month postoperative follow-up, lower limb and IVC ultrasounds showed no new acute thrombosis formation, no IVC obstruction, and no symptomatic PE.

### 3.3 Filter removal factor analysis

A correlation analysis of the two groups showed a relationship between filter retrieval and factors such as radiation dose, filter deformation, and whether the filter cranial anchor vertex penetrated the IVC wall. In the analysis of filter inclination, there was a correlation with cranial anchor vertex penetration distance and the transverse diameter of the IVC (Table 2).

**Table 2:** Correlation analysis between IVC filter retrieval and tilt.

Topic	Correlation Analysis of IVC Filter Retrieval		Correlation Analysis of Severe Inclination	
	*r*	*P*	*r*	*P*
Radiation Dose	0.322	0.033	0.154	0.317
IVC Deformation	−0.396	0.008	−0.171	0.266
>3 mm Vessel Wall Transgression	−0.362	0.016	0.171	0.266
Penetration Distance of Cranial Anchor Vertex	0.223	0.129	−0.369	0.014
Transverse Diameter of IVC	0.010	0.947	−0.324	0.032

Logistic regression analysis of factors influencing filter retrieval showed that filter deformation had a statistically significant difference, with an odds ratio (OR) of 0.069 (0.006, 0.828), indicating it as an independent risk factor for filter retrieval.

Logistic regression analysis of factors influencing filter inclination showed that the penetration distance of cranial anchor vertex and the transverse diameter of the IVC had statistically significant differences, with ORs of 0.667 (0.465, 0.958) and 0.843 (0.712, 0.998), respectively, indicating these as independent risk factors for filter inclination (Table 3).

**Table 3:** Logistic regression analysis of IVC filter retrieval and tilt.

Topic	Regression Analysis of IVC Filter Retrieval		Regression Analysis of Severe Inclination	
	OR (95% CI)	*P*	OR (95% CI)	*P*
Radiation Dose	1.001 (0.999,1.004)	0.361	-	-
IVC Deformation	0.069 (0.006, 0.828)	0.035	-	-
>3 mm Vessel Wall Transgression	0.000	0.998	-	-
Penetration Distance of Cranial Anchor Vertex	-	-	0.667 (0.465,0.958)	0.028
Transverse Diameter of IVC	-	-	0.843 (0.712,0.998)	0.048

## 4 Discussion

### 4.1 Results and significance

Due to the complications associated with long-term filter retention (25–28), which can have serious physiological and psychological impacts on both patients and their families (23, 29), this center places a high emphasis on the retrieval of retrievable filters. For conical filters whose retrieval hook attached to the wall, once the retrieval hook has penetrated the vein wall, continuing to pull the filter towards the proximal end will not correct the filter's inclination, nor align the filter's longitudinal axis with the retrieval sheath. Pulling the filter towards the proximal end may cause the retrieval hook to move further outside the vascular lumen or completely penetrate the vein wall, significantly reducing the chances of intraluminal retrieval. The filter retrieval method used in this study primarily involves pulling the conical filter towards the distal end, which not only moves the retrieval hook back into the lumen but also partially corrects the inclination of the filter. This is then combined with various advanced retrieval techniques to increase the chances of successful intraluminal retrieval. Among the 44 patients in the study, 37 successfully had their filters retrieved, increasing the intraluminal retrieval rate of conical filters whose hook attached to the wall to 84.1%, thereby effectively improving the retrieval rate.

In the study, factors potentially influencing filter retrieval were analyzed using logistic regression. The results showed a statistically significant difference in filter deformation, identifying it as an independent risk factor influencing filter retrieval. Therefore, during the filter retrieval process, it is important to avoid morphological changes in the filter's main pedicle and secondary filter strands, as these changes, especially in the secondary strands, are more likely to occur during the retrieval process. After such changes, the inclination angle of the filter may increase, or the radial support force of the filter may be enhanced, thus raising the risk of penetration by the retrieval hook or the inclined legs. Furthermore, these morphological changes may affect the implementation of advanced retrieval techniques. Therefore, in cases where filter deformation is detected, it is advisable to first restore the deformed filter pedicles before using advanced retrieval techniques to remove the filter. Previous studies have suggested that a penetration distance of filter cranial anchor vertex greater than 3 mm might indicate potential retrieval hook penetration (22), but in this study, no statistical difference was found between the penetration distance of filter cranial anchor vertex and filter retrieval.

In the study, factors influencing severe filter inclination were analyzed using logistic regression. The results suggested that the penetration distance of filter cranial anchor vertex and the transverse diameter of the IVC significantly affected severe filter inclination, making them independent risk factors for the occurrence of severe filter inclination. The IVC below the renal veins often appears elliptical, especially in individuals with a slimmer body type. For conical filters that make point contact with the IVC wall, during filter release, the main pedicles of the filter first contact the longitudinal axis of the IVC. The larger the transverse diameter of the IVC, the more likely it is that one or more of the filter's main pedicles will fail to contact the venous wall, leading to filter inclination.

It is important to note that this technique is applied only after the failure of multiple advanced filter retrieval techniques. The introduction of procedural sequencing may lead to complications such as filter deformation, increased hook penetration, and greater tilt angle during the use of these advanced retrieval methods, ultimately affecting the success rate of filter retrieval and resulting in biased outcomes. Therefore, when utilizing advanced filter retrieval techniques, excessive traction and pulling on the filter's stabilizing arms should be avoided to minimize surgical complications. Additionally, careful preoperative assessment of enhanced CT imaging results is essential for analyzing and determining the appropriateness and feasibility of each technique, which can ultimately improve filter retrieval rates.

Regarding surgical complications using this technique, one patient in this study experienced IVC rupture: intraoperative attempts to dilate the damaged IVC using a balloon were ineffective, but the patient's vital signs remained stable; the outer vascular sheath of the IVC was intact; and no significant contrast diffusion was observed. After conservative treatment, including anticoagulation therapy and 1-week bed rest, a follow-up CT scan revealed narrowing of the IVC at the ruptured site with attached thrombus, but no IVC obstruction (Figure 3). 1 other patient had a broken secondary filter strand. After retrieving the main body of the filter, a snare was used successfully to retrieve the broken secondary filter strand (Figure 4). The broken strand could also have been retrieved using forceps (14).

### 4.2 Combined use of other filter retrieval techniques

Loop Snare Technique: Once the retrieval hook penetrates the vein wall, this technique cannot achieve coaxial alignment. Pulling the filter towards the proximal end will cause the retrieval hook to embed further into the vein wall or completely penetrate it. Pulling the filter toward the distal end first can not only correct its angulation but also move the retrieval hook away from the venous wall, allowing the retrieval sheath to more easily dissect the endothelial tissue adherent to the hook, thereby facilitating filter retrieval.

Hangman Technique: This technique cannot be used when there is no gap between the filter neck and the IVC wall. Traction of the filter toward the distal end can create a gap between the filter neck and the venous wall, thereby increasing the success rate of the Hangman technique.

Forceps Technique: With an adjustable bending sheath and forceps, the retrieval hook can be captured directly, but the endothelial tissue covering the surface of the retrieval hook can prevent successful capture. The filter may be pulled toward the distal end to thin the endothelial tissue along the venous wall at the level of the retrieval hook and increase its tension. Combined with the use of grasping forceps to disrupt and dissect the endothelial tissue, this can gradually expose the retrieval hook, thereby enabling successful capture of the hook and retrieval of the filter.

Restoration of Deformed Filter Shape: When the secondary filter strands or balancing arms are curled or deformed, this technique should first be used to restore the filter to its normal configuration. It reduces the radial supporting force of a tilted filter and, during the application of various filter retrieval techniques, avoids interference from metal guidewires, thereby facilitating the implementation of these techniques.

### 4.3 Comparison with open surgical filter retrieval

Laparoscopic techniques are suitable for retrieving conical filters whose retrieval hook attached to the wall, especially for filters that have penetrated the vein wall. However, this approach has two limitations: ①It is difficult to confirm whether the retrieval hook has penetrated the vein wall. Literature suggests that if the cranial anchor vertex of the retrieval hook is more than 3 mm away from the vein wall, it is considered to have penetrated. However, in this study, approximately 59.1% of patients had the cranial anchor vertex of the retrieval hook more than 3 mm from the vein wall, and the filter was still successfully retrieved intraluminally using this method. This increased the intraluminal retrieval success rate to 84.1% after the advanced Loop snare technique failed. ②When the retrieval hook penetrates the vein wall, especially if it is located at the posterior wall of the IVC, it is more difficult to retrieve the filter using laparoscopic techniques. The filter's retraction hook capture technique of pull-assisted method, however, is not limited by the location of vein wall penetration.

Open surgical filter retrieval is suitable for all types of filters, particularly for long-term retained non-conical filters or those with serious complications. However, it involves longer surgery times, greater tissue damage, and longer recovery periods. The use of the filter's retraction hook capture technique of pull-assisted method reduces the need for open surgery, but it should be noted that using this new technique to retrieve such filters carries the risk of significant venous damage, which may require open surgery for vein wall repair.

### 4.4 Comparison with other relevant studies

Desai et al. (30) reported complications related to retrievable filters and methods for using advanced technologies for filter retrieval. Their approach involved using bronchial forceps to separate the endothelial tissue from the retrieval hook, followed by retrieval using either a retrieval set or bronchial forceps. This method is similar to the one used in this study, which also employed forceps to retrieve wall-attached conical filters. This method to retrieve filters is often effective for patients whose retrieval hook was encased in thrombus and thus cannot be captured. However, for filters where the retrieval hook had penetrated the vein wall, the forceps were unable to reach the hook and disrupt the endothelial tissue covering it. In such cases, the filter should first be pulled towards the distal end to generate a force that moves the retrieval hook back into the lumen. The local vein wall also experiences high tension, which aids in the forceps' ability to contact the retrieval hook and separate the endothelial tissue, successfully exposing the retrieval hook and increasing the chances of intraluminal retrieval.

Anzai (21) reported on 107 cases using advanced techniques for filter retrieval, including 4 cases using a double-sling technique, which is similar to the method used in this study. In their approach, forceps were used at the proximal end, while a wire loop was formed on the hook of the filter at the distal end, creating a bidirectional tension. However, this technique has some potential drawbacks: Due to endothelialization of the filter's pedicles, it may not be possible to form a wire loop. If a wire loop is formed and tension is applied at the distal end, the resulting cutting force may completely free the suspended pedicles from the vein wall. If the filter is not successfully retrieved at this point, further attempts to use a wire loop to suspend the free pedicles become difficult. In contrast, this study used forceps at the distal end, allowing for better control of the filter's position and the possibility of repeated capturing attempts. Once the filter's legs are freed from the vein wall, it becomes easier to capture the filter, which increases the chances of successful retrieval.

## Limitations

Several factors contribute to the variability in filter retrieval success, including the design characteristics of the filters and hooks, operator-dependent techniques, imaging discrepancies, and potential biases inherent in the study design.

Firstly, the design of conical filters, particularly the stabilizing arms, introduces resistance during distal retrieval, limiting the distance the filter can move. Additionally, variations in filter materials, stiffness, and deformability can further influence how far the filter can be displaced. The retrieval hook design, especially when featuring sharper and hollow tips, may result in deeper penetration of the vein wall, potentially complicating hook capture and retrieval.

Secondly, operator experience and technique play a significant role in the retrieval process. Proficiency in using the snare device and understanding the nuances of applying counter-traction is essential for successful retrieval. As such, operators with more experience tend to perform more efficient and precise retrievals. Given the steep learning curve associated with this procedure, variations in operator skill may impact the overall success rate.

Imaging discrepancies between portovenous CT and digital subtraction angiography (DSA) also introduce challenges. For instance, while CT may show greater hook penetration, DSA often fails to capture the full extent of filter tilt due to differences in projection angles. Both imaging modalities are also limited in their ability to accurately measure venous wall thickness at the hook location. As such, hook penetration exceeding 3 mm should not automatically be interpreted as a perforation of the venous wall.

Finally, the retrospective, single-center design of this study introduces potential selection bias. Filters with severe deformation or those that could have been successfully retrieved but were excluded for patient-related reasons were not included in the analysis. Additionally, the relatively small sample size and the fact that all procedures were performed by qualified operators at a single center limit the generalizability of these findings. A multi-center, larger cohort study is necessary to validate these results and reduce potential biases.

## Conclusion

In conclusion, the filter's retraction hook capture technique of pull-assisted method is effective in removing conical filters whose hook attached to the wall and successfully retrieving them intraluminally. This method avoids the severe risks associated with open surgery, with no cases of symptomatic PE observed, and can serve as a new adjunctive method for filter retrieval.

## Data Availability

The original contributions presented in the study are included in the article/Supplementary Material, further inquiries can be directed to the corresponding author.
